# Vaccine Confidence and Coverage among Medical Students at a Federal University in Brazil

**DOI:** 10.3390/vaccines12090993

**Published:** 2024-08-30

**Authors:** Ricardo B. Feijó, Jordana V. H. Bertotto, Amanda C. Pinto, Maria Eduarda T. G. Leal, Víctor M. de Souza, Vitória F. Sakai

**Affiliations:** 1 Departamento de Pediatria, Faculdade de Medicina, Universidade Federal do Rio Grande do Sul, Porto Alegre 90035-003, Brazil; amandapinto@hcpa.edu.br (A.C.P.); meleal@hcpa.edu.br (M.E.T.G.L.); victorsouza@hcpa.edu.br (V.M.d.S.); vsakai@hcpa.edu.br (V.F.S.); 2 Hospital de Clínicas de Porto Alegre, Porto Alegre 90035-903, Brazil; jhendler@hcpa.edu.br

**Keywords:** vaccines, vaccine coverage, medical students, vaccination coverage, immunization, medical education, health promotion, vaccine intervention, public health, vaccine barriers

## Abstract

Background: Declining vaccination coverage (VC) and vaccine hesitancy among medical students are global challenges. These challenges reflect individual and logistical barriers to a sufficient adherence toward essential vaccines for healthcare professionals, as well as presenting a need for educational strategies during undergraduate training. Methods: This is a prospective study for evaluating VC rates, sociodemographic associations, and the vaccine confidence among medical students at a federal university in Brazil. The data collection included questionnaires and individual analyses of the participants’ vaccination records. Results: A total of 237 medical students from all six years of an undergraduate program participated, of whom 124 (52.3%) had a vaccination record. Although the majority considered the vaccines to be “Completely Safe” (86.9%), the VC rates for complete vaccination schedules were relatively low, ranging from 87.9% (hepatitis B vaccine) to 3.2% (meningococcal B vaccine), including the vaccines from the National Immunization Program (NIP) and the private sector. Higher VC rates were found to occur among students in the final years of their undergraduate studies, in those from families with higher monthly incomes, and those from private secondary schools. Conclusions: Given the low VC rates among medical students, other factors in addition to vaccine confidence may be determinants, thus highlighting the importance of reviewing policies for the inclusion of priority groups in the NIP and in implementing educational interventions during undergraduate training.

## 1. Introduction

Recent years have seen a troubling decline in vaccination coverage (VC) and an increase in vaccine hesitancy worldwide, exacerbated by the COVID-19 pandemic. In Brazil, significant drops in VC were observed from 2016 to 2019, particularly in childhood immunizations. The pandemic worsened this decline due to physical distancing, limited mobility, and fears of transmission in health centers. Between 2020 and 2021, Brazil reported its lowest VC rates in history, including among healthcare workers, although data on vaccine hesitancy and coverage among medical students remain scarce [1,2].

Vaccine hesitancy, as defined by the WHO’s Strategic Advisory Group of Experts (SAGE), is a complex phenomenon influenced by specific contexts. It involves a delay in the acceptance or refusal of vaccination despite the availability of services. Based on the “3 Cs” model (complacency, confidence, and convenience)—later expanded to the “5 Cs”—vaccine hesitancy is affected by factors such as trust in vaccine effectiveness and safety, the competence of healthcare professionals, and the motivations of policy-makers [3,4,5,6].

Although healthcare professionals, including medical students, are trained to promote vaccination and understand the risks of vaccine-preventable diseases, individual confidence in vaccines can vary. This variation can reflect discrepancies in VC and acceptance, regardless of the academic knowledge gained during medical training.

The vaccination of medical students is an important but underexplored topic. Existing studies often rely on indirect data from questionnaires rather than confirmed vaccination records. This is crucial for preventing personal infection and the spread of diseases in clinical settings. Understanding how vaccine hesitancy impacts this group, given their acquired knowledge and perceptions of vaccine safety, is essential [7,8,9].

### 1.1. International and National Context

Globally, the VC among medical students varies, with higher rates for mandatory vaccines and lower rates for recommended ones [10,11,12]. In Europe, rates are generally higher for vaccines considered essential (e.g., BCG, diphtheria, tetanus, polio, and hepatitis B) and required by law for practice entry. In contrast, rates are lower for vaccines that are only recommended (e.g., influenza, pertussis, varicella, measles, and rubella), which are not legally required but are strongly recommended to protect healthcare workers and patients from vaccine-preventable infections [13]. In Brazil, non-compliance with routine vaccinations is common, and while some universities have shown improvements through vaccination campaigns, gaps still remain [14,15,16,17].

### 1.2. Teaching and Attitudes

Medical students’ education on immunization is often insufficient. Many students report limited knowledge on vaccines, which can contribute to vaccine hesitancy [18,19,20,21]. Factors such as exposure during practical training and reliable information sources influence attitudes toward vaccination [22,23,24,25,26]. Additionally, vaccination recommendations and policies vary, with Brazil’s National Immunization Program providing broad coverage but lacking specific guidelines for medical students [27,28,29,30,31,32].

During medical school, students learn about the importance of vaccines and the risks of vaccine-preventable diseases (complacency) and have easy access to vaccines (convenience). However, their confidence in vaccines may vary, making it essential to assess this confidence among students in training.

Understanding medical students’ perceptions and attitudes toward vaccines is key to developing targeted interventions that improve vaccine education and confidence early in their training. This study aims to assess vaccination coverage by reviewing vaccination records and evaluate vaccine confidence among medical students at a federal university in Brazil, exploring how confidence and sociodemographic factors influence this population.

## 2. Materials and Methods

This prospective study was conducted in 2023. The researchers approached all of the students enrolled in the Medicine course at the Federal University of Rio Grande do Sul (UFRGS) in Brazil with the aim of evaluating VC rates, possible sociodemographic relationships, and vaccine confidence.

After approval by the institution’s Ethics and Research Committee (CAAE 6811.4223.9.0000.5347), the research team (composed of 2nd and 5th year medical students) underwent a three-month training period on immunization. This training included the following:

- Lectures on immunization (basic immunology, the mechanism of action of vaccines, the development and production of vaccines, adverse effects, etc.);
- The presentation of vaccination schedules of the National Immunization Program (NIP) of the Ministry of Health and the Brazilian Immunization Society (SBIm);
- Workshops to discuss clinical cases and training to evaluate vaccination records.

Data collection instruments were developed, and all protocols were recorded on a digital platform (Google Classroom) such that the research team could continuously refer to and work with them.

In the next stage, over a period of three months, direct contact was made with the students from all six academic years (*n* = 801), and the research protocol was presented to them. There was face-to-face contact during the theory classes of the course (the academic cycle of which lasts from the 1st to 4th years), and virtual contact via social networks (in the internship cycle, which lasts from the 5th to 6th years). All the individual data for access to students (face-to-face and virtual) were obtained after analysis and advice from the UFRGS School of Medicine’s Undergraduate Committee. Informed consent was obtained from all the subjects involved in the study.

Of the 237 students who responded to the questionnaires (i.e., 29.8% of those who enrolled), 124 sent in their vaccination records for the team to evaluate. All the vaccination records were evaluated and checked individually in accordance with the recommendations of the Brazilian Immunization Society (SBIm) and the Ministry of Health for each age group and for health professionals (which included vaccines available in the public and private networks).

After analyzing the data, an individual report was sent to each participating student. These reports detailed the students’ incomplete or absent vaccination schedules, which of the vaccines were available through the NIP (or private sector), as well as the scientific material on the reference vaccination calendars. Furthermore, individual advice was made available via email or social networks with the team.

Certain vaccines recommended by both the NIP and the Brazilian Immunization Society are only available for free in the public network for certain age groups. Therefore, once the age range defined by the NIP is exceeded, these vaccines are then only available in the private sector. This is the case for the HPV4 vaccine (which is available from the NIP for adolescents under 15 years of age), the meningococcal C/ACWY vaccine (both menC, which is available up to 5 years of age, and meningococcal C/ACWY booster, which is available for those between 11 years of age and under 15 years of age), and hepatitis A (which is available only for children at 15 months of age in a single dose).

The vaccination schedules were classified as follows:

- Complete;
- Incomplete;
- Absent.

The vaccination coverage was analyzed using complete vaccination schedules in accordance with the specific indications for each vaccine (primary and booster) for the age of the population studied (e.g., MMR: two doses; hepatitis B: three doses; meningococcal B: two doses; meningococcal ACWY; and dT: primary + booster). A previous diagnosis of an immunopreventable disease was considered if it was recorded (e.g., varicella). A complete COVID-19 schedule was considered with three doses, the last of which was in the current year (2023).

Individual sociodemographic data, as well as data on vaccine safety opinion, vaccine confidence, and vaccine registration, were collected. Monthly family income was assessed using the national monthly minimum wage (MW), which was approximately USD 270 in 2023.

In Brazil, some of the vaccines administered by the National Immunization Program (NIP) are still physically recorded on a vaccination card provided by the Ministry of Health from birth. In the private sector, vaccines administered may be recorded either on the same card or on a different specific card. Although the registration of vaccinated persons in basic health units has been computerized since 2019, due to the aim of integrating data at the national level (including vaccines from the private network), there are still difficulties in accessing the history of all individual vaccinations. In addition, the physical record has great documentary value [29,33,34]. For this reason, the data collection in this study regarding vaccination records was based exclusively on the physical records submitted individually and sent directly to the team for analysis.

The data were analyzed using the SPSS program (v. 29) and started with descriptive analysis, which included the mean and standard deviation for parametric quantitative variables, the median and interquartile range for nonparametric variables, and the frequency/percentage for categorical variables. In addition, comparisons between the groups were conducted using nonparametric tests such as Mann–Whitney or Kruskall–Wallis for quantitative variables, and Chi-square or Fisher’s exact test for categorical variables.

We did not calculate a sample size because the entire population of students was targeted in this study, inviting all to participate. The power was 100% to test whether the total confidence proportion differed from 50%. This was based on a 5% significance level, a sample size of 237, and an expected total confidence rate of 30%.

## 3. Results

### 3.1. Demographic Characteristics

There were 237 participants (29.8% of the total number enrolled in the medical course) with a mean age of 24.6 years (standard deviation 4.47 years), of whom 134 (56.5%) were female and 103 (43.5%) were male. With regard to high school education, 141 (59.5%) reported having been educated in private schools and 91 (38.4%) in public schools. There was a distribution in all the categories studied with respect to the students’ monthly family income, with 87 (36%) reporting a monthly family average of more than ten minimum wages (MWs), 53 (22.4%) between five and ten MWs, 47 (19.8%) between two and five MWs, and 26 (11%) with less than two MWs (Table 1).

### 3.2. Vaccine Confidence and Registration

When assessing the level of safety of the vaccines, the majority of the survey participants (*n* = 206, 86.9%) indicated that they thought vaccines were “Completely Safe”, while 28 (11.8%) thought vaccines were “Partially Safe”. Only 1.2% of students (*n* = 3) responded that vaccines were “Completely or Partially Unsafe” (Table 1).

Regarding the existence of a vaccination card, 192 (81%) stated that they had a vaccination card, while 24 (10.1%) reported that their card had been lost or misplaced, and 21 (8.9%) did not know whether they had a vaccination card. Among the group that answered the questionnaires, 124 (52.3%) sent in their vaccination records according to the requested criteria, which were then analyzed individually (Table 2).

### 3.3. Vaccination Coverage

The vaccination coverage was generally relatively low for both the vaccines offered by the NIP and those that are available only through the private sector.

Among the vaccines offered free of charge by the NIP, the VC rates, in descending order, were as follows: 87.9% for hepatitis B; 75% for measles, mumps, and rubella (MMR); 69.4% for yellow fever; and 64.5% for diphtheria and tetanus.

The different schedules were also evaluated specifically for the COVID-19 vaccine, where it was found that 66.1% had incomplete schedules, 30.7% had complete schedules, and 3.2% were without schedules.

For the vaccines available only in the private sector (given the students’ current age group), the VC rates were even lower than those for the vaccines offered by the NIP. It was found that 43.5% of schedules were complete for the HPV vaccine, whereas only 26.6% were complete for hepatitis A, 22.6% for varicella, 17.7% for the meningococcal C/ACWY vaccine, and only 3.2% for the meningococcal B vaccine.

### 3.4. Relationships between the Socioeconomic Data and Vaccine Confidence

There was a statistically significant relationship between family income and vaccine confidence, highlighting a positive association between a family income of the equivalent of five minimum wages or more and a “Completely Safe” perception of vaccine safety. On the other hand, there was an association between a family income of the equivalent of less than five minimum wages and a “Partially Safe” perception of vaccine confidence (*p* = 0.039).

### 3.5. The Relationships between Both Socioeconomic Data and Vaccine Confidence and Vaccine Coverage

The median number of complete schedules per student was only 1, with an interquartile range of 0 to 5. Students from private schools had a median of 2 complete vaccination schedules (interquartile range: 0–5), compared to those from public schools (with a median of 0 (interquartile range: 0–3), and this was for both vaccines available in the private network and for those offered by the National Immunization Program (i.e., hepatitis A, hepatitis B, varicella, meningococcal C and ACWY) (*p* = 0.005).

The perception of vaccines as “Completely Safe” was not correlated with higher vaccination coverage rates. A significant proportion of students with absent or incomplete vaccination schedules reported a “Completely Safe” perception of vaccines (86.5%). Additionally, 49% of students who perceived vaccines as “Completely Safe” did not have a complete vaccination schedule. It is important to note that the number of students with a “Completely or Partially Unsafe” perception of vaccines was limited, which may affect the generalizability of these results.

There were no significant variations in the median number of complete vaccination schedules in relation to gender, place of birth (urban or rural), or family income.

### 3.6. Relationships between Socioeconomic Data and Vaccination Coverage for Specific Vaccines

**Figure 1:**
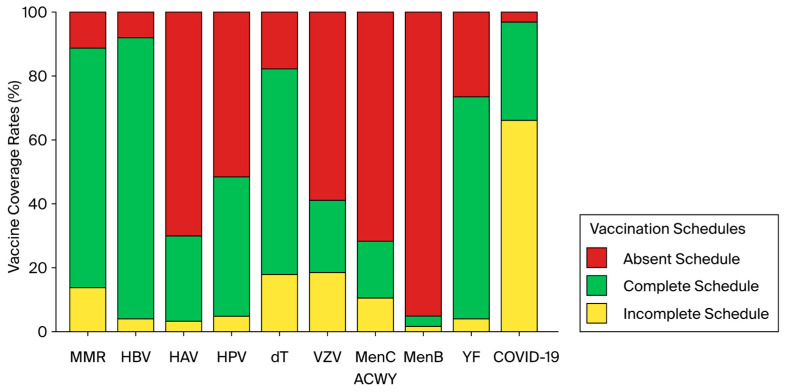
Percentage of complete, incomplete, and absent vaccination schedules for each analyzed vaccine. MMR—measles, mumps, and rubella; HBV—hepatitis B; HAV—hepatitis A; HPV—human papillomavirus; dT—diphtheria and tetanus; VZV—varicella zoster virus; MenC or Men ACWY—meningococcal conjugate vaccine (MenC) or meningococcal ACWY vaccine; MenB—meningococcal B vaccine; YF—yellow fever; and COVID-19—COVID-19 vaccine.

For specific vaccines, there was a wide variation between the vaccination coverage of each schedule, as shown in Figure 1. Female sex and a history of private school education were associated with higher vaccination coverage. For COVID-19 vaccination, the majority of students with complete schedules were female (68.4%), while among those identified with incomplete schedules, 54.9% were male (*p* = 0.006). Of the complete MMR schedules, 60.2% were administered to girls, while of the absent schedules, 71.4% were administered to boys (*p* = 0.045). For the yellow fever vaccine, 64% of the complete schedules were administered to girls, while 66.7% of the absent schedules were administered to boys (*p* = 0.003).

When comparing the vaccination of public and private school students for the hepatitis A vaccine, 87.5% of complete schedules were from private school students, while 46.4% of incomplete schedules were from public school students (*p* < 0.001). For the hepatitis B vaccine, 74.7% of complete schedules were from private school students, while 100% of incomplete schedules were from public school students (*p* = 0.012). For the ACWY vaccine, 89.5% of complete schedules were from private school students, while 36.7% of incomplete schedules were from public school students (*p* = 0.019). In the case of the varicella vaccine, 96% of complete schedules were from private school students, while 43.5% of absent schedules were from public school students (*p* < 0.001).

In contrast, for the COVID-19 vaccine, 71.8% of incomplete schedules were from private school students, while 50% of complete schedules were from public school students (*p* = 0.045).

### 3.7. Analysis of the Sociodemographic and VC Data via the Stage of Academic Training

Adherence to the survey was higher among students in the first four years of university (i.e., the academic cycle), totaling 83.2% (*n* = 198) of the questionnaires returned. However, the majority of students in the internship cycle (the fifth and sixth years) sent in their vaccination records (*n* = 29; 72.5%), which was in contrast to the 95 (48%) students in the academic cycle (*p* = 0.008).

There was no difference in age, sex, or urban or rural origin between the academic and internship cycle groups. Regarding primary education, 82.5% of the internship cycle group studied at a private school, while 35.5% of the academic cycle group studied at a public school (*p* = 0.03). There was no difference found for secondary education. 

Regarding family income, 82.9% of the internship cycle group had an income above five minimum wages, and 37.6% of the academic cycle group had an income below five MWs (*p* = 0.032). There was no difference found in the vaccine confidence between the groups.

The academic cycle group had a median number of complete schedules of 0 (with an interquartile range of 0 to 4), while the internship cycle group had a median of 4 (with an interquartile range of 0 to 6) (*p* = 0.002). The VC was similar between groups for the following vaccines: COVID-19 (complete schedule: 30.6%); dT (64.5%), yellow fever (69.4%), hepatitis B (87.9%), HPV (43.5%), meningococcal B (3.2%), MMR (75%), and varicella (22.6%). The vaccines that showed a difference between the groups were hepatitis A (44.8% of the internship cycle group had a complete schedule and 75.8% of the academic cycle group had an absent schedule, *p* = 0.037) and ACWY or meningococcal C (34.5% of the internship cycle group had a complete schedule and 77.9% of the academic cycle group had an absent schedule, *p* = 0.014).

## 4. Discussion

### 4.1. Main Results

This study is one of the first to evaluate, based on individually confirmed vaccination records, the vaccination coverage among medical students in Brazil. It highlights the considerable demographic and socioeconomic diversity among the participants, thereby reflecting a representative sample of the medical students in Brazil. The results showed significantly low vaccination coverage among the students, even for vaccines made freely available by the Brazilian National Immunization Program (NIP). Despite the high vaccine confidence among the students (86.9%), vaccination coverage remained below ideal levels. This suggests that vaccine confidence may not be the only determinant of low vaccination coverage among medical students, thus indicating the need to explore other possible needs and interventions to improve the VC in this population. A multivariate analysis, including Poisson regression with robust variances for vaccine confidence outcomes, was conducted, but no significant or relevant results were observed.

### 4.2. Vaccine Confidence

Most students expressed confidence in the safety of vaccines; however, 11.8% of the students perceived vaccines as only partially safe, and 1.2% perceived vaccines as unsafe, thus indicating a need for vaccine safety education for a significant proportion of students.

Additionally, socioeconomic factors were related to vaccine confidence, showing a positive association between household income above the equivalent of five minimum wages and the perception of vaccines as “Completely Safe”, while an income below the equivalent of five minimum wages was associated with the perception of vaccines as “Partially Safe”. This finding may be attributed to several interrelated factors. Individuals with a higher income generally have better access to accurate health information and higher levels of education, leading to greater confidence in the safety and efficacy of vaccines [35]. They also have better access to health services, including vaccination sites, which reduces logistical barriers [36]. Moreover, these individuals tend to have more consistent interactions with healthcare providers, which increases trust in medical recommendations [37]. Economic stability allows them to focus more on the long-term benefits of vaccination without the immediate economic pressures faced by lower-income individuals [35]. Stronger social support networks and greater awareness of the risks of vaccine-preventable diseases, as well as the benefits of vaccines, also contribute to a higher uptake [37,38].

### 4.3. Vaccination Coverage and Associated Factors

The variation in vaccination coverage found in this study reflects not only the availability of vaccines through the NIP, but also adherence to the vaccines available through the private health network. While some vaccines, such as hepatitis B, had high coverage rates, others that are available only through the private sector had much lower coverage. Such a finding highlights the influence of socioeconomic factors on vaccine coverage.

#### 4.3.1. The Influence of Secondary Education

Significant disparities in vaccination coverage, which were identified through the presence of a significantly higher VC than those from public institutions, were observed among the medical students from private schools compared to those from public institutions, thus demonstrating the influence of socioeconomic status on vaccination adherence.

Access to health services has a significant impact on vaccine uptake, particularly among higher-income individuals (who generally have better access to medical care), which reduces logistical barriers to vaccination. Studies show that metrics such as having health insurance, a usual place of medical care, and recent visits to the doctor are positively correlated with higher COVID-19 vaccination rates [39]. These factors facilitate access to vaccination services, reduce the time and effort required to get vaccinated, and increase trust in health professionals. Higher-income individuals are more likely to have health insurance and a regular healthcare provider, which not only improves access to vaccines, but also ensures that they receive accurate and timely information about vaccination [40,41]. In addition, routine medical checkups, which are more common among higher-income individuals, are associated with less vaccine hesitancy and higher vaccination rates [41]. These individuals also face fewer economic barriers to accessing health services, such as transportation costs or the need to miss work, thus allowing them to prioritize preventive health measures, including vaccination.

#### 4.3.2. Influence of Gender

Coverage was higher among women for the COVID-19, measles–mumps–rubella, and yellow fever vaccines. Women tend to have higher vaccination coverage than men due to a combination of factors, including access to health services, proactive health-seeking behavior, and targeted public health initiatives. Women generally have more frequent interactions with health professionals, particularly through reproductive health services such as gynecological examinations and prenatal care, which provide more opportunities for vaccine recommendations and administration [42,43]. Women who visit an obstetrician/gynecologist are more likely to receive vaccine recommendations (e.g., the HPV vaccine) [42]. In addition, women often demonstrate greater health awareness and are more proactive in taking preventive measures, with studies showing that those who are more health conscious are more likely to initiate and complete vaccination series [44,45].

On the other hand, men may face other barriers to vaccination, such as less frequent recommendations from health professionals, as well as cost and logistical challenges (such as scheduling appointments), which also make it difficult to adhere to vaccination [42,43,44,45,46,47]. Many men underestimate the risk of vaccine-preventable diseases, particularly with regard to HPV, and the norms of masculinity that value self-sufficiency may reduce the need to seek preventive care [48,49,50,51]. Overcoming these barriers requires targeted educational campaigns, a greater involvement of health professionals, and strategies to minimize logistical and financial barriers.

#### 4.3.3. Vaccination against COVID-19

Vaccination against COVID-19 has revealed significant challenges, with a significant proportion of students having incomplete vaccination schedules. COVID-19 vaccination coverage is one of the most studied worldwide. A systematic review and meta-analysis found that the worldwide prevalence of COVID-19 vaccination among medical students was 61.9% [52]. In comparison, the data collected in our study showed that only 30.6% of students had completed their COVID-19 vaccination, while 66.1% had incomplete schedules. Vaccination rates were higher among females and public school students. This particularly applied to the COVID-19 vaccine, where students from public schools had higher coverage rates than those from private schools.

#### 4.3.4. Other Vaccines

In addition to COVID-19, our results showed variable vaccination coverage for other vaccine-preventable diseases. While HPV vaccination uptake among medical students in southern Italy was 55.5% [24], only 43.5% of the students in our study had completed HPV vaccination. Similarly, we found a 75% coverage for measles vaccination compared to the 59.5% among unprotected Italian students [53]. These comparisons highlight regional differences in vaccine uptake among medical students, thereby suggesting the influence of contextual and educational factors.

### 4.4. Influence of Training Stage

The stage of medical training influenced the likelihood of vaccination coverage among the students in this study, which is a finding that is in line with international studies showing higher vaccination rates in more advanced stages [25,26]. These results highlight the importance of educational approaches and targeted interventions throughout the academic cycle to promote immunization and increase vaccine confidence. Targeted actions can improve the awareness and acceptance of vaccines, as well as preparing future health professionals to effectively promote public health.

### 4.5. Influence of Vaccination Policies

The low vaccination coverage among medical students may reflect the lack of more comprehensive inclusion in the NIP, indicating the need to review existing policies to ensure high vaccination coverage among medical students. Similarly, medical schools should review their strategies for teaching and promoting immunization among their students, which could be achieved via considering the importance of individual and collective protection from the beginning of undergraduate studies.

### 4.6. Challenges beyond Vaccine Confidence: Structural and Demographic Barriers to Vaccination Coverage

Despite the high rates of vaccine confidence, vaccination coverage may remain low due to several additional factors contributing to vaccine hesitancy. Structural and logistical barriers, such as limited vaccine availability, distribution issues, and access difficulties, significantly impact vaccination efforts [54]. Geographical disparities and socioeconomic factors, such as residing in disadvantaged areas or regions with high ethnic diversity, are linked to lower vaccine uptakes [55]. Additionally, gaps in education and understanding can lead to underimmunization, even in communities with low hesitancy levels [54]. Health system factors, such as health worker strikes and distances to vaccination centers, also pose significant barriers [56]. Furthermore, demographic factors, including age, race, and political affiliation, affect vaccination rates, underscoring the complexity of improving vaccination coverage beyond tackling mere hesitancy [57,58].

### 4.7. Limitations

Although the dissemination and resources used (face-to-face and digital) were extensive, student participation was particularly low (approximately 30%), which may indicate a lack of interest in the topic, as well as inadequate outreach to students. Similarly, the need to provide a physical or digitized vaccination record may have discouraged greater participation in the survey.

Although the sociodemographic characteristics of the students are similar to the majority of federal public universities, specific social and cultural characteristics may limit the generalizability of the results presented.

The vaccination records were documented over two decades, which may have affected the accuracy and completeness of the data due to macroeconomic changes and other factors over time. We carefully reviewed the records to ensure their integrity, including only clear and complete data in our analysis. We believe that, despite these potential limitations, the records provide a valuable foundation for our analysis, representing the most comprehensive data available at present.

## 5. Conclusions

This detailed study of vaccination coverage among medical students in Brazil revealed significantly low VC rates, even in the face of high vaccine confidence. Our analysis highlighted that vaccine confidence is not the only factor determining the low rates observed, thus indicating the need for additional interventions (especially during the undergraduate course). In addition to the influence of socioeconomic aspects, the failure to include medical students in the priority groups of the National Immunization Program suggests a gap in current policies, as well as a call for a review of strategies to ensure adequate protection of students and the communities in which they will work professionally.

## Figures and Tables

**Table 1:** Vaccine confidence, demographic characteristics, and vaccination coverage rates in medical students at the Federal University of Rio Grande do Sul (UFRGS), Brazil.

	Vaccine Confidence			Total (%) ^#^
	Completely or Partially Unsafe	Partially Safe	Completely Safe	
Sociodemographic Data	*n* = 3 (1.2%)	*n* = 28 (11.8%)	*n* = 206 (86.9%)	*n* = 237
Gender				
Female	2 (66.7%)	11 (39.3%)	121 (58.7%)	134 (56.5%)
Male	1 (33.3%)	17 (60.7%)	85 (41.3%)	103 (43.5%)
High School				
Private School	0 (0%)	18 (64.3%)	123 (59.7%)	141 (59.5%)
Public School	3 (100%)	10 (35.7%)	78 (37.9%)	91 (38.4%)
Monthly Family Income (MW) ^##^				
Up to 2 MWs (<USD 540)	1 (33.3%)	5 (17.9%)	20 (9.7%)	26 (11%)
2 to 5 MWs (USD 540–1350)	0 (0%)	7 (25%)	40 (19.4%)	47 (19.8%)
5 to 10 MWs (USD 1350–2700)	1 (33.3%)	2 (7.1%)	50 (24.3%)	53 (22.4%)
More than 10 MWs (>USD 2700)	0 (0%)	8 (28.6%)	79 (38.3%)	87 (36.7%)
Registered Vaccination Records	*n* = 3 (100%)	*n* = 14 (50%)	*n* = 107 (51.9%)	*n* = 124 (52.3%)
Vaccines (Complete schedules) ^###^				Vaccination Coverage Rate (%)
Measles/Mumps/Rubella (MMR) *	2 (66.7%)	10 (71.4%)	81 (75.7%)	93 (75%)
Hepatitis B *	2 (66.7%)	12 (85.7%)	95 (88.8%)	109 (87.9%)
Hepatitis A **	0 (0%)	3 (21.4%)	30 (28%)	33 (26.6%)
HPV ***	2 (66.7%)	7 (50%)	45 (42.1%)	54 (43.5%)
dT*	2 (66.7%)	9 (64.3%)	69 (64.5%)	80 (64.5%)
Varicella **	0 (0%)	1 (7.1%)	27 (25.2%)	28 (22.6%)
Meningococcal C or ACWY ****	0 (0%)	4 (28.6%)	18 (16.8%)	22 (17.7%)
Meningococcal B **	0 (0%)	1 (7.1%)	3 (2.8%)	4 (3.2%)
Yellow Fever *	2 (66.7%)	9 (64.3%)	75 (70.1%)	86 (69.4%)
COVID-19 *				
Complete Schedule	2 (66.7%)	2 (14.3%)	34 (31.8%)	38 (30.7%)
Incomplete Schedule	1 (33.3%)	12 (85.7%)	69 (64.5%)	82 (66.1%)
Absent Schedule	0 (0%)	0 (0%)	4 (3.7%)	4 (3.2%)

^#^ the total may vary in some categories due to the absence of responses or the “prefer not to answer” option; ^##^ MW (minimum monthly wage in Brazil) in 2023: ≈USD 270; ^###^ according to the criteria of the Ministry of Health and the Brazilian Immunization Society, Brazil; * available in the National Immunization Program (NIP); ** available in the private sector; *** available in the NIP for <15 years (HPV4) and in the private sector for >15 years (HPV9); and **** available in the NIP for <15 years and in the private sector for >15 years.

**Table 2:** Information on the vaccination records (i.e., vaccination cards).

Vaccination Card	n	%
I do not know if I have my card	21	8.9%
I do not have my card (lost or misplaced)	24	10.1%
I have my card (printed or digital)	192	81%
Total	237	100%

## Data Availability

The data that support the findings of this study are available from the corresponding author, R.B.F., upon reasonable request.
